# Microbial growth and adhesion of *Escherichia coli* in elastomeric silicone foams with commonly used additives

**DOI:** 10.1038/s41598-023-35239-9

**Published:** 2023-05-26

**Authors:** Ingrid Rebane, Hans Priks, Karl Jakob Levin, İsmail Sarigül, Uno Mäeorg, Urmas Johanson, Peeter Piirimägi, Tanel Tenson, Tarmo Tamm

**Affiliations:** 1 Institute of Technology, University of Tartu, Nooruse 1, 50411 Tartu, Estonia; 2 Institute of Chemistry, University of Tartu, Ravila 14a, 50411 Tartu, Estonia; 3 Estelaxe OÜ, Parksepa, Estonia

**Keywords:** Microbiology, Engineering, Polymer chemistry, Surface chemistry

## Abstract

Silicone is often used in environments where water repellency is an advantage. Contact with water promotes the adhesion of microorganisms and biofilm formation. Depending on the application, this may increase the possibility of food poisoning and infections, the material's degrading appearance, and the likelihood of manufacturing defects. The prevention of microbial adhesion and biofilm formation is also essential for silicone-based elastomeric foams, which are used in direct contact with human bodies but are often difficult to clean. In this study, the microbial attachment in and the retention from the pores of silicone foams of different compositions is described and compared to those of commonly used polyurethane foams. The growth of the gram-negative *Escherichia coli* in the pores and their leaching during wash cycles is characterised by bacterial growth/inhibition, adhesion assay, and SEM imaging. The structural and surface properties of the materials are compared. Despite using common antibacterial additives, we have found that non-soluble particles stay isolated in the silicone elastomer layer, thus affecting surface microroughness. Water-soluble tannic acid dissolves into the medium and seems to aid in inhibiting planktonic bacterial growth, with a clear indication of the availability of tannic acid on the surfaces of SIFs.

## Introduction

Silicone is a well-researched material with a broad range of applications. Nevertheless, its antibacterial properties in specific applications still raise questions. In medical applications, silicone-based foams (SIFs) are primarily used as prostheses^1^ and modern wound dressings^2,3^, constantly contacting body tissue and body fluids. As environmental humidity and water promote biofilm formation by the adhesion of microorganisms, such conditions undoubtedly increase the possibility of infections. Another rapidly growing field of application for SIFs is cushioning (seats, mattresses, gaskets), where occasional contact with body fluids, food, and liquids is highly probable. Cushioning foams often have an open-celled structure, which allows the penetration of air, fluids and microorganisms. As biofilm formation also degrades the appearance of the material and increases the likelihood of manufacturing defects^4,5^, the prevention of microbial adhesion and biofilm formation on silicone materials is an important subject, independent of their application.

Poly(dimethylsiloxane), e.g. PDMS-based polymers, commonly known as silicones, are not inherently antibacterial. Additives such as catalysts, including platinum nanoparticles^6^, and other low molecular weight species incorporated between polymer chains or grafted onto the polymer backbone may give silicone antibacterial activity^7–9^. Low surface tension and, therefore, high hydrophobicity is reported to be one of the primary reasons PDMS is prone to protein adsorption and bacterial adhesion^10,11^. For example, Busscher et al. compared *Candida albicans and C. tropicalis* and found that the more hydrophobic the surface of the microorganism, the more it is inclined to adhere to a silicone surface^4^. Although the gram-negative bacteria *Escherichia coli* have both hydrophobic and hydrophilic regions in its outer membrane layer, its surface is generally considered hydrophilic (the contact angle for wetting is reported to be in the range of 16.7°–24.7°)^12,13^. It is generally understood that the adhesion of microorganisms depends on hydrophobic interactions between the bacterial cell and the polymer surface^13^.

Seeking to suppress the adhesion of hydrophilic bacteria onto a hydrophobic surface, increasing surface hydrophilicity^10,14,15^ is often proposed as a possible solution. The adhesion of *E. coli* onto silicone catheter is shown to decrease by 32% by grafting antimicrobial peptide and polyvinylpyrrolidone on cured PDMS, or even up to ~ 95% by using vinyl-modified methyl cellulose^16^ and, using carboxymethyl chitosan and polydopamine resulted in a ≥ 90% reduction in *E. coli* adhesion^15^. Also, grafting acrylates^7^ to silicone rubber (*Pseudomonas*, catheter) effectively suppresses non-specific protein adsorption and cell adhesion, suppressing hydrophobic recovery of the surface. One of the most recent works by McVerry et al. shows a successful one-step hydrophilic surface modification in ambient conditions and under UV light to create a zwitterionic polymer polysulfobetaine and perfluorophenylazide network onto silicone surface^17^. The reported antibacterial activity was due to the formation of the surface hydration layer in the presence of the hydrophilic coating.

The surface modifications mentioned above are successfully conducted for monolithic silicone, allowing it to process the material's surface uniformly. However, it becomes much more challenging to graft the highly porous and low-density open-celled SIFs throughout the structure. Submerging large air-filled volumes would be cumbersome and time-consuming. Also, when moulding, a partial skin forms during the manufacturing process. Most importantly, the hydrophobic nature is instead favoured to deter water from its surface and pores, especially in cushioning and insulation applications. Functionalising its surface with hydrophilic groups would revoke its water-deterring property, thus acting as a sponge-like material.

To maintain the material's hydrophobic nature, it is possible to add local antibacterial sites in the form of particles, thus inhibiting the growth of microorganisms. These particles may partially surface during the thinning of pore walls in the curing and foam-formation process but remain embedded in the material. Such a fabrication method is based on the polymerisation and dispersion of components and avoids the wearing of antibacterial additives.

To apply antibacterial properties to silicone, incorporating different silver species (nanoparticles, salts, ions) has been one of the leading research directions for many years^18–21^. In conjunction with hydrophobic silicone, wound dressings are found to benefit in extracting bacteria from wounds and act bactericidal simultaneously^2^. Inorganic nanoparticles, Ag, ZnO, and TiO_2_ show high antibacterial efficacy against *E. coli*, but their use in effective concentrations alters mechanical properties significantly^20^. For large volumes of material, several low-cost additives and fillers could be incorporated into the foam matrix. For example, tannic acid^22–24^, shungite^25^, chitosan^26^, mica, and zinc-based^27^ have shown antibacterial activity.

The microbial activity on silicone foams has been investigated mainly for commercial wound dressings^2,19^ through qualitative (i.e. zone inhibition) and quantitative (bacterial viability assay) tests. In a powdered form, a shake-flask method has been used to test the antibacterial activity of peroxide-cured high-density SIFs, where the antimicrobial effect lies on toxic additives leaking from the (post-)cured foam^28^. For different wound dressings, zone inhibition test results have been compared with the hydrophobicity of the porous material surface^2^. For unfoamed silicone elastomers, an untreated porous membrane has also been shown not to reveal any bacteriostatic or bactericidal activity^29^.

Recently, a standardised method, ISO 23641:2021^30^ was published for testing flexible cellular polymers based on the shake flask method and could be used as a guide for evaluating the antibacterial effectiveness. In some cases, a simplified process could be applied as effectively.

This study focuses on the antimicrobial activity in polysiloxane foams with different mineral and organic additives, which are readily available and thus industrially feasible. For comparison, polyurethane-based (PU-, or PUR-) foams and their antibacterial activities are assessed, as PU is the most widely used material in the previously mentioned applications and many more. There is extensive research on PU-foam and its antimicrobial additives (in wound dressings^19^, membranes^31^, composites^31^ and coatings^31^), but for SIFs, many research questions are still unanswered as this field is constantly evolving. More importantly, both materials are applied as cushioning layers in seats and mattresses. As elastomeric silicone foams are increasingly gaining interest in cushioning, vibration dampening, insulating^32^ and medical applications, tuning their antimicrobial properties add value to their already superb nature.

## Results and discussion

### Antibacterial effect on the growth medium

**Figure 1:**
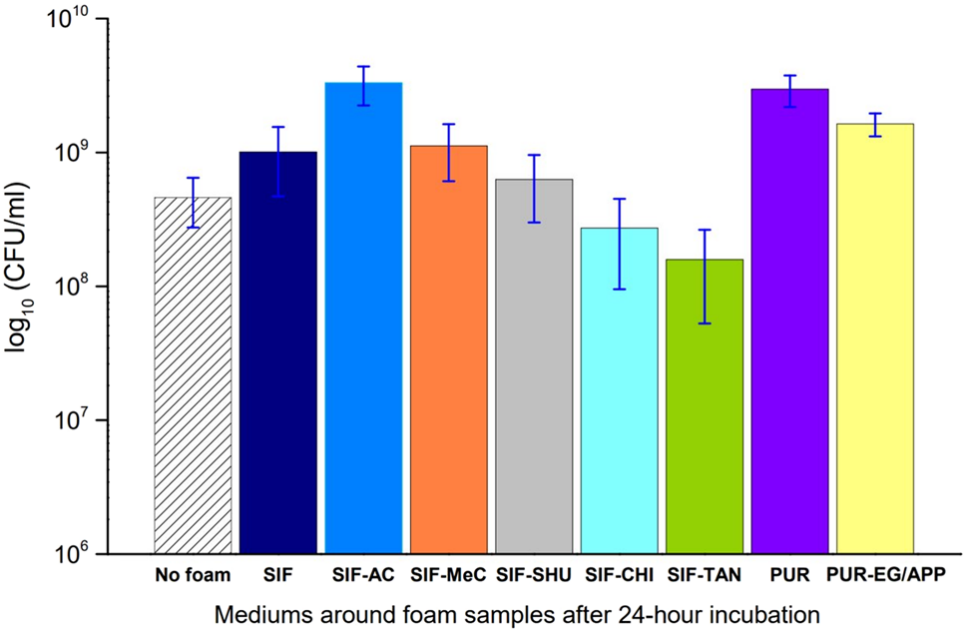
The variations in *E. coli* concentrations in the growth medium surrounding the cube samples, and in the medium without a foam sample. A significant difference from a standard SIF can be seen for SIF-CHI (silicone foam with chitosan additive) and SIF-TAN (silicone foam with tannic acid additive). Also, both PUR-based foams show higher *E. coli* concentrations in the growth medium compared to other SIFs (except SIF-AC with active carbon additive). *SIF-MeC -* silicone foam with methyl cellulose additive, *SIF-SHU -* with shungite additive, *PUR-EG/APP -* polyurethane foam with exfoliated graphite and ammonium polyphosphate additive. The abbreviations used in this figure and properties of the prepared foams are summarized in Table 1.

This experiment shows how the different additives in the foam and the difference in base material affect bacterial growth in the inoculation suspension within 24 h at 25 °C. The resulting concentrations of *E. coli* in Luria-Bertani medium (LB broth) surrounding the test cubes are depicted in Fig. 1. As the foam cube is immersed in the medium, it is freely permeable to the bacteria and the carrying medium. *E. coli* concentration is expected to differ from the pure, cube-less growth medium and around cubes proposing an antibacterial effect. For this, the same tests were conducted for a pure inoculation mix (No foam) and a standard silicone foam without antibacterial additives (SIF) for the control experiment.

The results show that the concentration of *E. coli* (c[CFU]) in the growth medium was the highest around SIFs with activated carbon additive (SIF-AC). Compared to the pristine (additive-free) SIF, the *E. coli* concentration (CFU/ml) in the surrounding growth medium of SIF-AC is threefold higher. This significant difference in c[CFU] suggests that the hydrophilic activated carbon additive increases the concentration of the growth medium around the foam cubes. Activated carbon is described as an adsorbent to bind molecules from liquids by van der Waals forces, causing a higher concentration of adsorbate at the interface than in the bulk fluid^33^. Therefore, the effect on c[CFU] in growth media around SIF-AC could result from the increased attachment of bacteria on the foam's pore surface, which is also seen on the SEM (Scanning Electron Microscope) images (section Bacterial population formations). Although methyl cellulose's hydrophilic and water-soluble nature would allow it to diffuse from the polymer composite and dissolve in LB, its inclusion in the SIF matrix (in SIF-MeC) does not have a significant effect on inhibiting bacterial growth in the medium compared to pristine SIF. A foam-less growth medium resulted in 1/3 logs lower c[CFU] than with the SIF foam specimen, which suggests that the presence of a SIF foam cube increases the *E. coli* /LB concentration in 24 h at 25 °C.

The inoculation suspensions around SIF-SHU, with shungite additive, resulted in lower c[CFU]-s than for pristine SIF, although it is essential to notice that the standard deviation ranges overlap; hence the difference may not be significant. As shungite is reported to have antibacterial properties in an aqueous medium, we expected an effect on c[CFU]^25^. SIFs that significantly hinder the growth of E. coli at 25 °C are SIF-CHI and SIF-TAN. Compared to pristine SIFs, the hydrophilic chitosan additive in SIF-CHI seems to have a distinguishable effect in inhibiting bacterial growth in the medium. As chitosan is water-insoluble, its dissolution from the polymer is not expected. Its protrusion from the surface to some degree or availability from the cut sides of the foam is possible. From previous research by Qin et al*.,* the water-insoluble chitosan shows an inhibitory effect against *E. coli* due to the water acting as an acidic medium^34^. For SIF-TAN, during the inoculation period, a visually detected discolouration of the growth medium indicates tannic acid (TA) leakage into the medium. The leakage is further promoted by cutting the sample and exposing TA to the solution. As TA is a highly soluble molecular substance, vibrational diffusion of the molecules from a hydrophobic cross-linked polymer network and dissolution into an aqueous medium is possible^35^. The SIF-TAN foam sample resulted in the best antibacterial effect among other additive-doped foams in this test, resulting in 0.5 logs lower c[CFU] than for pristine SIF.

In general, cell adhesion to hydrophobic surfaces has been previously described by McVerry et al. as due to the nonpolar nature of PDMS and the significant increase in antibacterial effect when its surface is modified to hydrophilic^17^. As the surface of PUR is naturally hydrophobic^36^ but less hydrophobic than silicone, bacterial adhesion and growth in the surrounding medium are expected when there is a possibility to use the material's surface for attachment and multiplication. Contact angle measurements (see Supplementary data S1) show that although both SIF and PUR are hydrophobic (Θ > 90°), the water droplet wets PUR and PUR-EG/APP surface more efficiently than SIF and SIF-AC surfaces. We have observed the wetting to increase in time due to the porous nature of the foams.

Despite a 0.5 log difference in c[CFU] between SIFs and PURs, we also need to consider the structure of the foam. Near similar foam densities, SEM micrographs reveal comparably larger voids in the PURs, which suggests that the bacteria have less surface area to adhere to and, therefore, to multiply. Also, the vigorous movement of the medium during shaking could hinder the adhesion of bacteria. The corresponding images comparing foam structures can be found in the Online Appendix S1 of this manuscript.

### *E. coli* adhesion, growth, and leaching from porous foam

**Figure 2:**
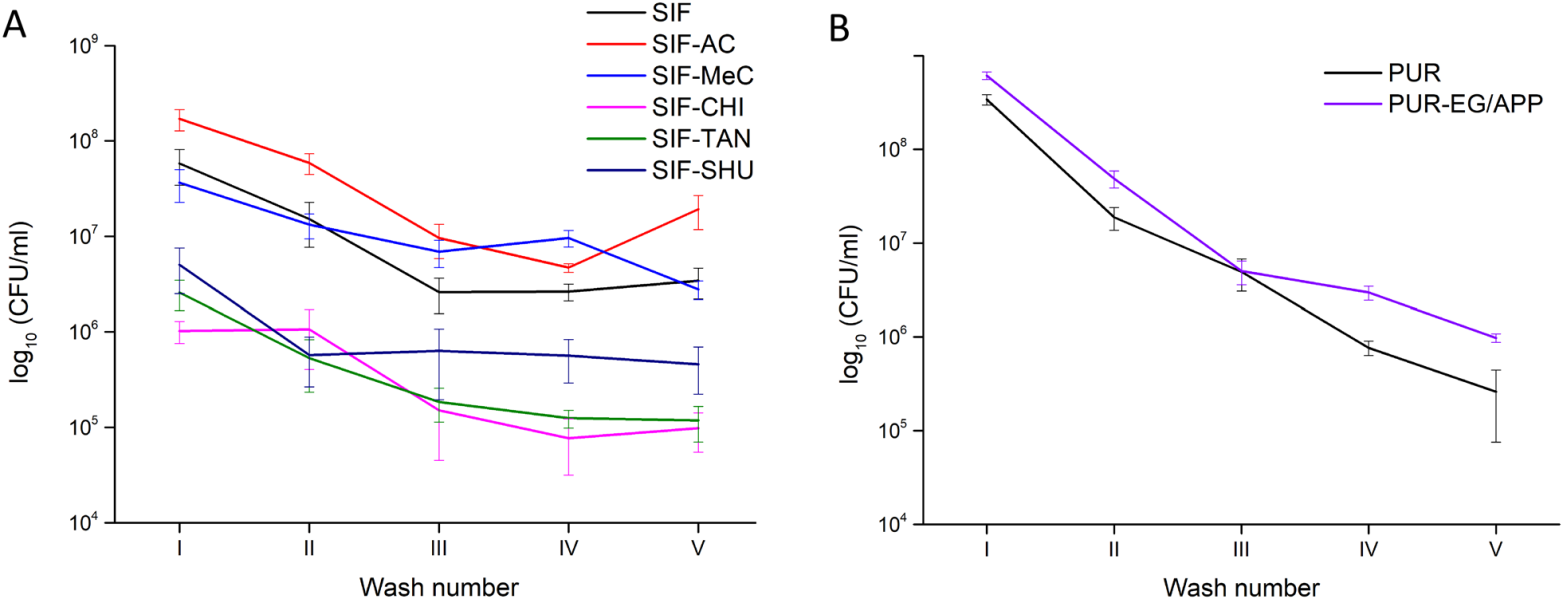
Extraction of the bacteria from cubes via wash cycles: concentrations of *E. coli* in the sequential washout mediums (CFU/ml of PBS). Silicone-based foams (**A**) are separated from the polyurethane foams (**B**) due to the difference in pore structure—the latter have significantly larger voids and the exchange of the medium is favored.

Samples, which were inoculated in E.coli/LB for 24 h at 25 °C (180 rpm), were washed in 1 × PBS for five sequential cycles to assess the adhesion-detachment behaviour of bacterial cells from the pores (planktonic cells) and pore walls (adhered cells). The following results in Fig. 2A,B indicate how different materials affect bacterial adhesion and growth and whether the antibacterial additive has any effect inside the foam. From the first wash (wash I), the c_I_[CFU] leaching is in correlation with the c_24h_[CFU] being highest for polyurethanes and lowest for SIF-TAN/SIF-CHI. Comparing the c[CFU] of wash mediums shows that a relatively large portion of bacteria leaches out with the 1st wash indicating the majority to be planktonic, e.g. floating inside the foam. The sum of bacteria washed out from the foam would not give us the overall bacteria inside the material, as not all cells are released within five washes. Still, the subsequent washes (from wash II–V) reveal more bacteria present in total than in the surrounding medium.

A steeper slope at the beginning of Fig. 2 depicts the extraction of loose (planktonic) viable cells. We suggest that the shallow plateau at the end of the curves (washes III, IV, and V) could indicate the slow detachment of adhered cells from the pores (Fig. 2A). The steepest curves from washes I-III project well the highly porous structure of PUR and PUR-EG/APP, allowing faster passage for the suspension. Contrary to PURs, the bacteria are more difficult to extract from the SIFs. Possible mechanisms include the more probable adhesion to the more hydrophobic surfaces and the obstruction of the smaller pore size, which lowers the rate of liquid exchange during washes. As the SIF-TAN had the lowest *E. coli* concentration in the surrounding growth medium after 24 h of incubation, the early plateauing curve is expected due to the initially fewer bacteria present and the antibacterial effect due to the leaking of TA into the solution.

**Figure 3:**
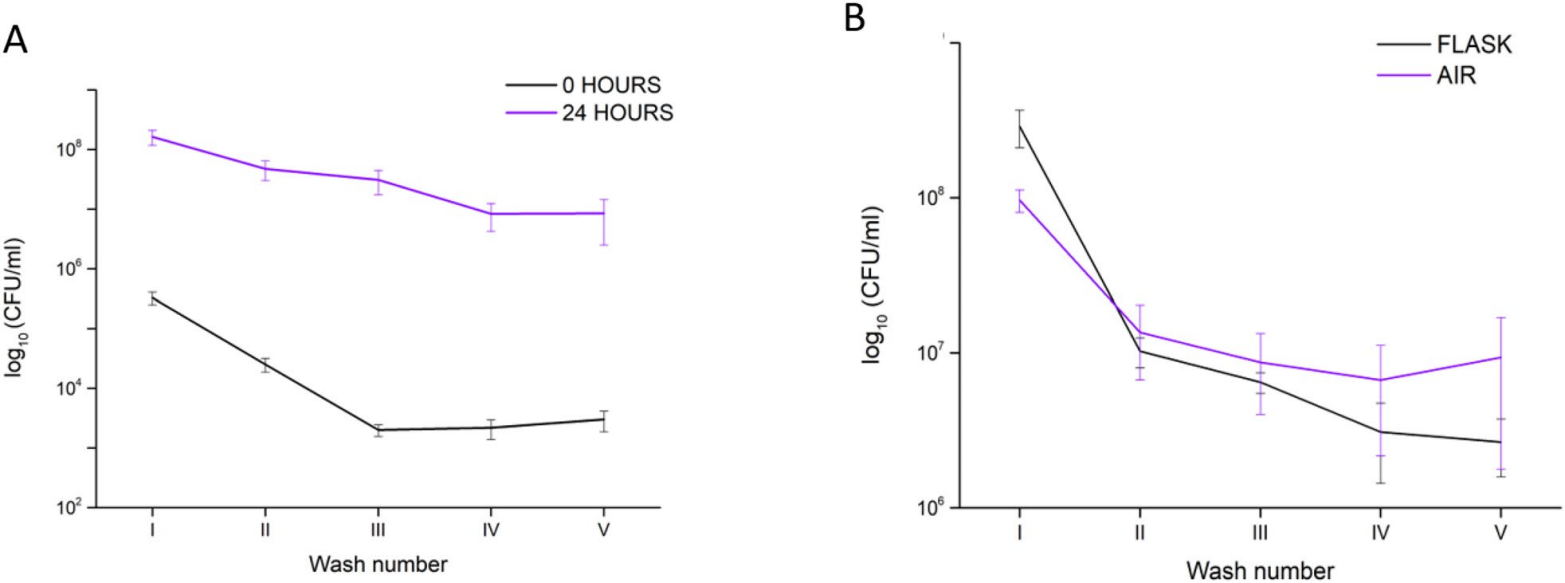
(**A**) Release of bacteria from pristine SIF foams at different durations of inoculation: foam inoculated for 24 h and foams washed immediately after the inoculation step (no antibacterial additives). (**B**) This experiment shows the differences in concentrations arising from different growing conditions. In experiments conducted in ‘AIR’, there is no excess broth and no additional aeration/shaking. After the 24-h period, inoculated standard SIFs were analyzed for *E. coli* concentrations (CFU/ml).

We were also interested in whether there was significant bacterial growth in the foam cube or whether the results from washes were merely the effect of adhesion. One possibility was to compare if the attachment/adhesion of *E. coli* is affected by the inoculation duration. The differences between short-term (0 h, immediate washout) and long-term (24 h) inoculations were analysed, and the summary is depicted in Fig. 3A.

As before, the steeper curve allows us to assume that more bacteria are loose (planktonic) and less adhered. As expected, a more extended period has allowed the bacteria to grow but also to interact with the surface of the foam material. The wash curves after 24-h incubation period show slower elimination from the pores than for immediately washed out bacteria. Such plateauing could indicate gradual detachment behaviour from the hydrophobic material.

To illustrate the growth effect in low humidity conditions, as well as in lack of nutrients, we conducted a test in a closed environment at 25 °C without additional broth or shaking, followed by five subsequent washes (AIR test, Fig. 3B). Compared to the previously used method in this research, which is incubation in a flask and LB medium in excess, the elimination of bacteria from the foam samples results in somewhat similar curves of washout concentrations, indicating that when the bacteria have sufficient time to grow and multiply, there is enough time to adhere to the material's surface.

### Structure and surface of foams

**Figure 4:**
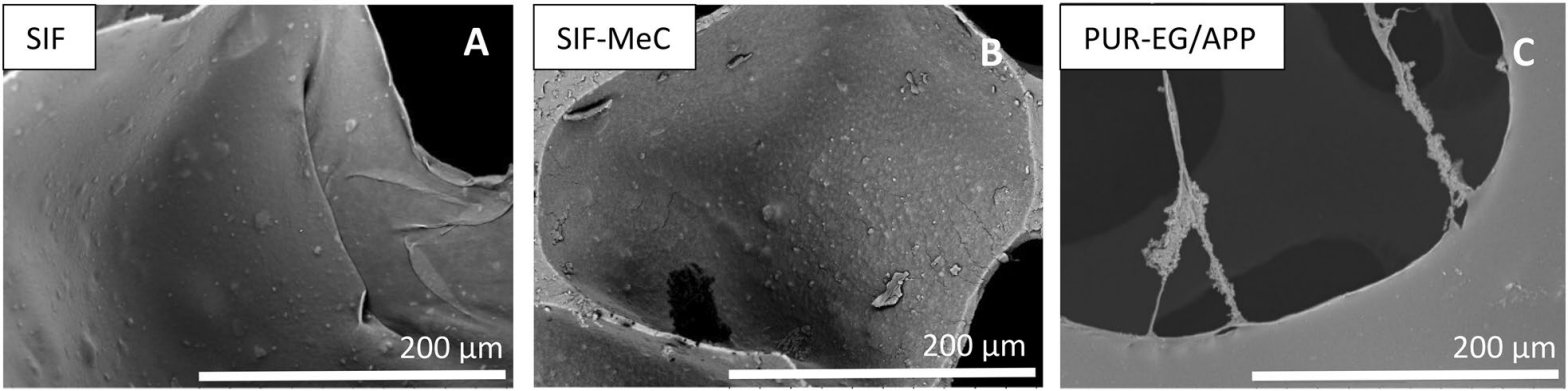
For a SIF without specific antibacterial additives, distinguishable general filler particles inside the foam walls are covered with thin polymer layer due to increased surface tension (image **A**). For SIF-MeC, additive increases the surface roughness, still remaining under polymer layer (image **B**). Smooth surface and hollow structure of PUR-EG/APP offers less surface to adhere to (image **C**).

All silicone foams used in this study are open-cell elastomeric polysiloxane-based foams. The foams were prepared using non-antibacterial general fillers distributed into the pre-polymers. Such filler particles are visible under the polymer layer (see Fig. 4A) of a pristine SIF. The surface with antibacterial additives differs only slightly from the pristine SIF visually by surface roughness and particle distribution, as seen in the middle image (Fig. 4B, SIF-MeC). Although general fillers used in SIFs compositions were not used for the standard PUR and PUR-EG/APP, the EG or APP particles used in this composition were not visibly detected protruding the surface (Fig. 4C).

Antibacterial-purposed additives make up a relatively large portion of the pre-mixture, varying from 0.3 to 5.0 wt%. The spatial dimensions of the particles compared with the pore wall thickness should allow them to surface/protrude during the blowing process and the consequent wall thinning. Therefore, we expect them to affect bacterial growth upon partial or direct contact with bacteria when the additive can disturb the cell membrane causing cell lysis and death^15^.

**Figure 5:**
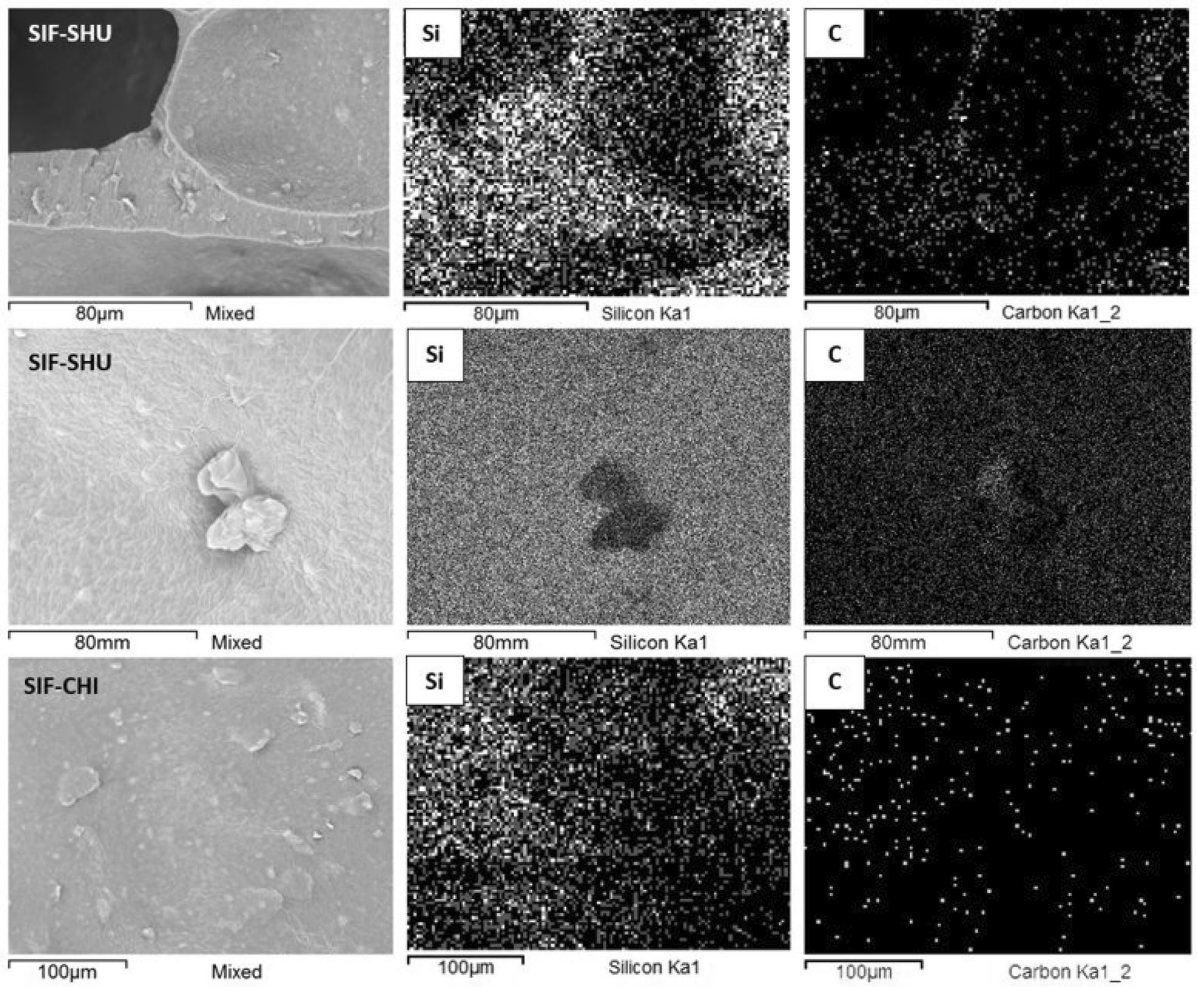
The SEM–EDX analysis elemental maps of SIF-SHU foam skeletal cross-section and pore surface show uniform Si distribution, indicating that silicone is present on the surface of filler particles. The elemental map of the cross-section reveals particular carbon-dense areas, which match the filler particles seen in SEM images. Si elemental map shows silicone is present on the filler particles to some extent. For SIF-CHI, the elemental compositions were as follows: C 61.2, O 6.3, Si 32.5 weight%, and C 76.7, O 5.9, Si 17.4 atomic%. For SIF-SHU, the elemental compositions were: C 48.9, O 28.8, Si 22.3 weight%, and C 61.0, O 27.0, Si 11.9 atomic%.

However, it is possible that due to the synthesis conditions and resulting foam parameters (wall thickness, additive content and particle dimensions, but most importantly, surface tension), these particles do not surface fully, and the majority remain covered with a thin polymer layer. To confirm this phenomenon, we performed EDX analysis on the SIFs surface by focusing on 20 different areas to acquire the surface's elemental composition. The distribution of elements (Si, C) is shown on the elemental maps obtained by the SEM–EDX technique (Fig. 5).

Compositional imaging results suggest that the particles are well incorporated into the silicone pre-polymers, leaving tangled polymer chains on the surface of the additives. For example, carbon-dense shungite areas in the polymer matrix are depicted by obtaining the EDX spectrum. Details of the EDX spectra of the SIFs surface values measured in atomic and weight percentages are listed under images in Fig. 5. Although the elemental composition of additives used is mostly C and H, the SEM–EDX results for SIF-CHI also show a low nitrogen concentration on the pore surface.

#### Bacterial population formations

**Figure 6:**
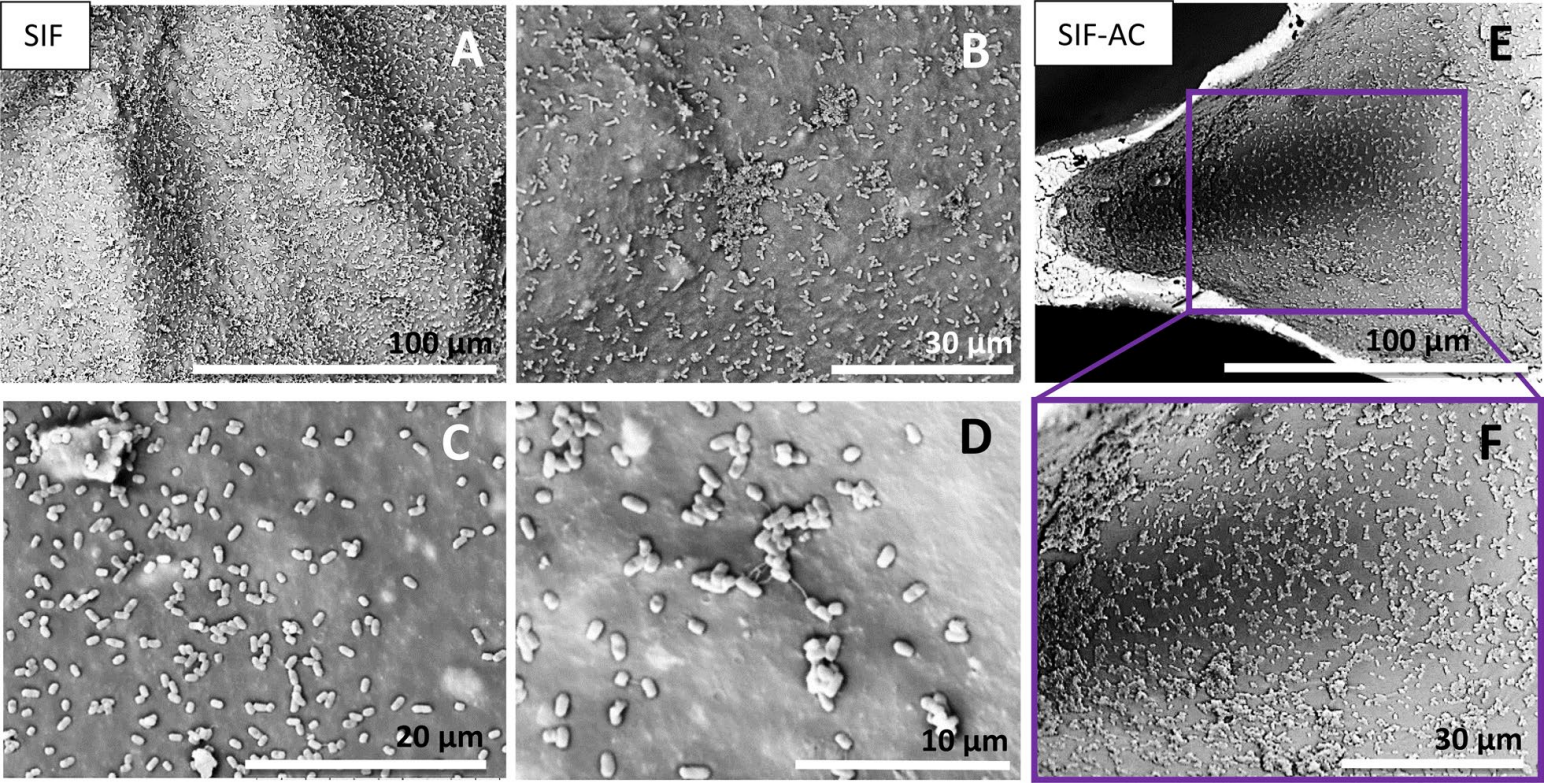
Even distribution of *E. coli* on the surfaces of SIF (images **A**–**D**) and SIF-AC (**E**,**F**). Multiple *E. coli* are present on top of the polymer, below which the polymer is covering the filler particles (images **B**,**C**). The formation of pili is visible on pristine SIF (image **D**). For SIFs with activated carbon, the bacteria form agglomerated areas which is distinctively different formation from standard SIF surfaces.

Amongst all observed samples, after the 24-h incubation period (without washouts), the bacteria tend to locate in different structural formations on the surface of the SIFs. An even distribution of adhered *E. coli* is only observed on pristine SIFs and SIF-AC (Fig. 6), which suggests that there is less or no disturbing effect for the bacterial adhesion favoured by the hydrophobic surface characteristic of silicone. The bacteria have also adhered to the sites where fillers are present—without any visible distinguished behaviour on or around it (Fig. 6, upper center image).

Activated carbon with a high surface area is considered hydrophilic (the higher the surface area, the higher the hydrophilicity) and with high adsorption capacity^33^. We found bacteria spread evenly on the pore surfaces in all foams with AC additive and some larger colonies/agglomerations on single sites. The formation of pili, which are mainly responsible for attachment during conjugation, allowing binding to solid surfaces, is evidence of suitable conditions for the gram-negative bacteria to share genetic information and multiply (Fig. 6, bottom center image). The pili were rare in these spread formations but more probable in colony cluster formations.

**Figure 7:**
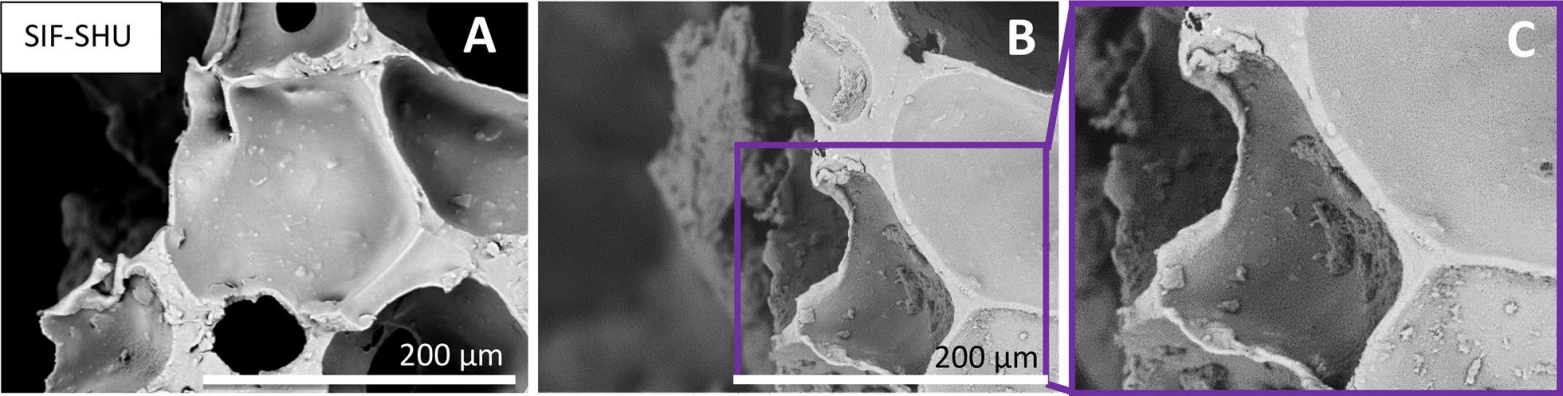
Due to the dissolution of components from shungite, bacteria are found only in the outmost pores of the SIF-SHU sample (images **B**,**C**). In the outmost pores, some agglomerated bacterial formations are found. Images (**A**) and (**B**) are replicates of the different areas of the same sample, and image (**C**) is magnification from image (**B**).

In addition to similar formations on SIF and SIF-AC, we noticed distinctive behaviour in groups of water-insoluble additives (shungite, chitosan) and water-soluble additives (tannic acid, methylcellulose). Although shungite has carbon in different modifications and is insoluble, it contributes some dissolving components that pose antibacterial properties^25,37^. SEM analysis reveals that the bacteria are found only in the outmost pores of the SIF-SHU sample leaving the inner pores almost bacteria-free (Fig. 7). Leaking of such components from the skeleton would explain the absence of adhered bacteria in the foam. Initially, the concentration of dissolved particles in the inoculation suspension is lower around the cube where the concentration of *E. coli* is relatively high (30.5 ml of total inoculation suspension, of that < 2.7 ml in the cube).

**Figure 8:**
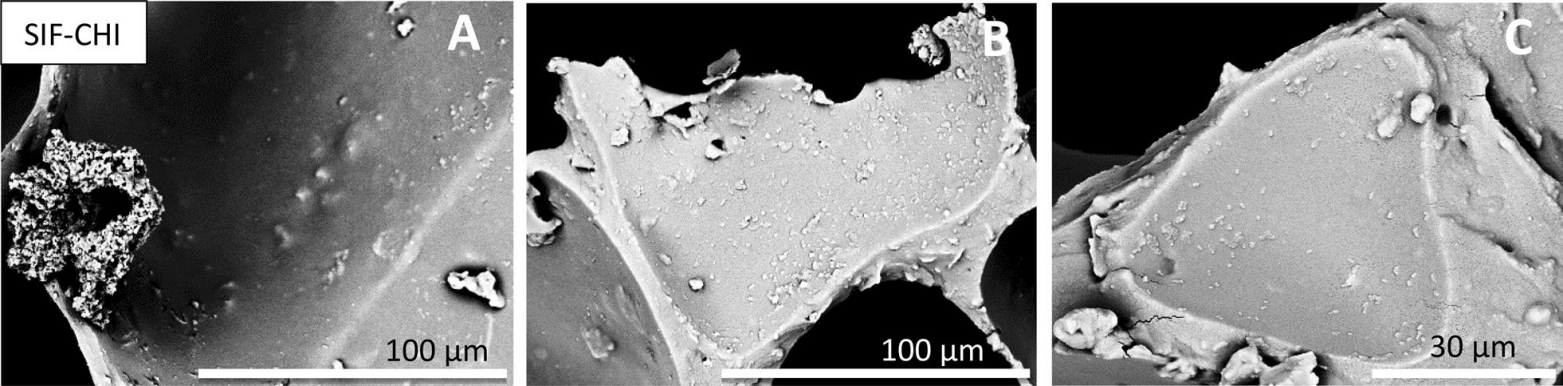
Chitosan-doped foams (SIF-CHI) showed an uneven distribution of *E. coli* on the surface of the pores. On images (**A**–**C**) some areas are occasionally populated, and some clean without certain formations. Images (**A**–**C**) are replicates of the same observed sample.

Samples with insoluble chitosan additive (SIF-CHI) show an uneven distribution of adhered *E. coli* (Fig. 8). We found only a few microorganisms in some pores, and pores closer to the sides of the sample were unevenly populated. Again, some bacteria were as large clusters/agglomerates were present (Fig. 8, image A), which may have been planktonic forms before the supercritical extraction. No such distribution, as was seen for pristine SIF (Fig. 6) and SIF-AC (Fig. 6), was found.

**Figure 9:**
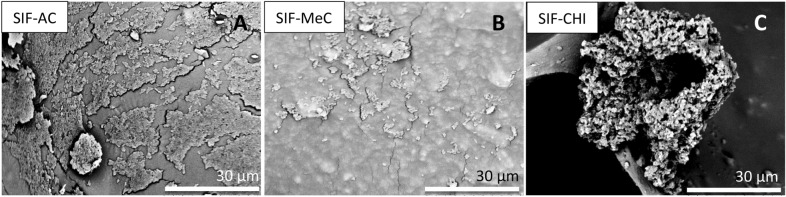
The formation of different structures on surfaces of SIFs with hydrophilic additives: bacteria as layered formations in SIF-AC (**A**), occasionally distributed adhered bacteria in SIF-MeC on a surface with visibly increased roughness (**B**), and single large c clusters of bacteria in the pores of SIF-CHI (**C**).

In this research, we have found that the antibacterial effect of methylcellulose (in SIF-MeC) on the surface of the material is similar to chitosan (in SIF-CHI) (Fig. 9B,C), but its antibacterial effect is negligible in the growth medium (Fig. 1). The latter indicates that the methylcellulose and chitosan do affect the adhesion of bacteria in the pores of silicone foam.

**Figure 10:**
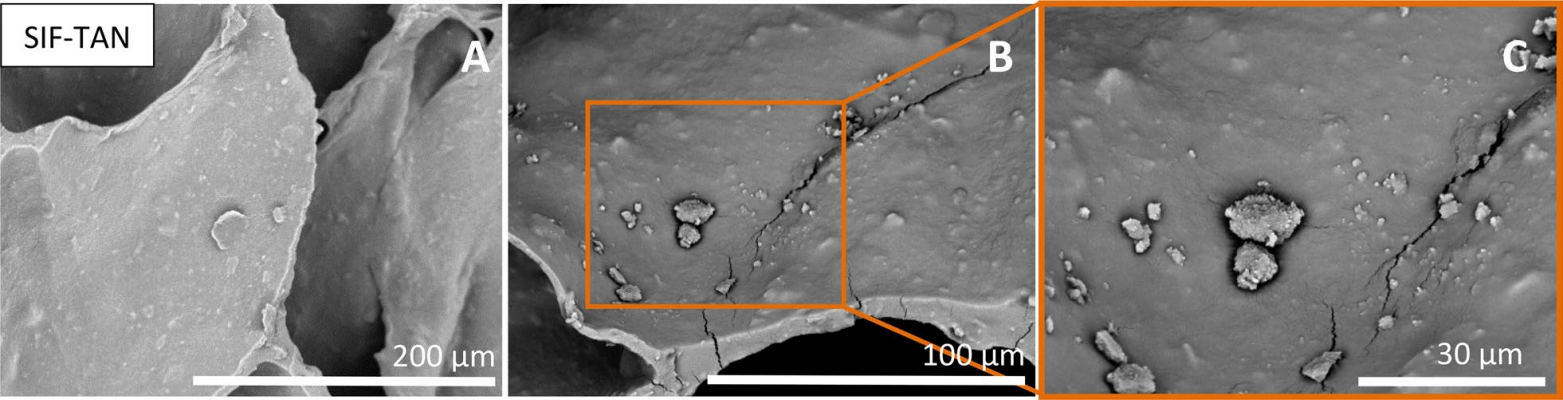
Absence of bacteria on the surface of a pore in SIF with a 0.5 wt% TA additive. In images (**B**) and (**C**), no *E. coli* is present, but residues of filler particles can be found on the pore surface. Images (**A**) and (**B**) are replicates of the different areas of the sample, and image (**C**) is a magnification of image (**B**).

Interestingly no bacterial adhesion as described above was visible on pore surfaces in the foam with the tannic acid (TA) additive. During the incubation period with vigorous aeration (180 rpm), highly soluble TA (solubility: 2850 g/L water, 1.7 mol/L) leaked from the foam, detectable from the discolouration of the suspension from yellow to brownish-yellow. As the samples were freeze-cut before inoculation, some TA particles may have become loose and uncovered. In the respective SEM images (SIF-TAN), we did not find any bacteria on the pore walls (Fig. 10). In SIF-TAN, pores are interconnected, and considering the size of the *E. coli* (2–5 µm), bacteria were able to free float throughout the void space of the foam cube along with the liquid medium. As there is no visible surface colonisation, we assume that the bacteria leaked during washes were floating and multiplying in the inoculation suspension (e.g. staying planktonic). The planktonic lifestyle of microorganisms in similar conditions has also been suggested by Tan et al.^38^.

**Figure 11:**
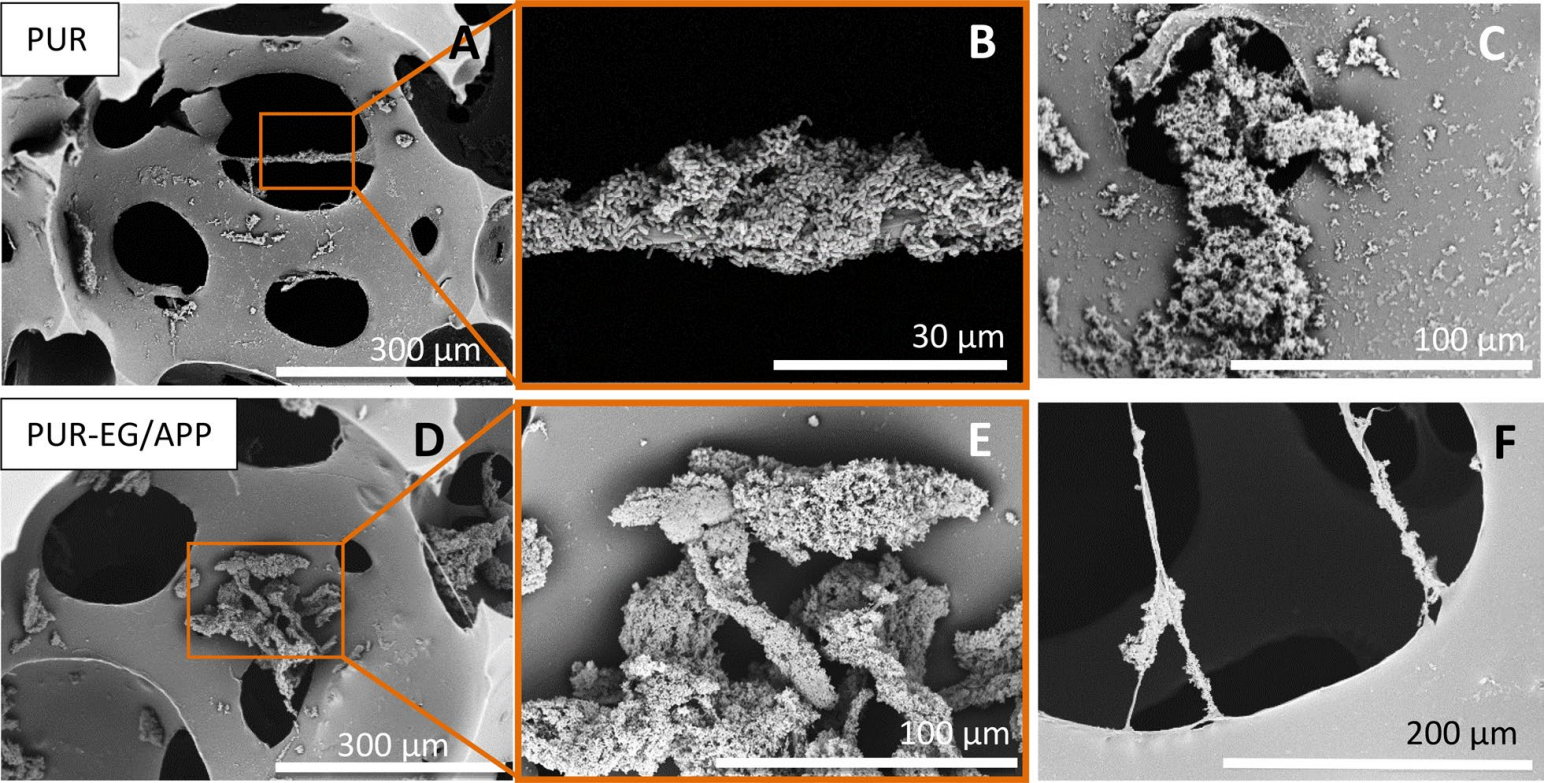
Polyurethane foams (PUR, images **A**–**C**, and PUR-EG/APP, images **D**–**F**) have a distinguished hollow structure. The majority of the bacteria has adhered to the thread-like formations across the pore voids (images **A**,**B**,**F**). For PURs in general, multiple large bacterial formations of *E. coli* are found which are not prevalent in SIFs.

Compared to the SIFs, the analysed low-density PURs (PUR and PUR-EG) had significantly less surface area to adhere to, and the structure is hollower (Fig. 11, upper row). Interestingly, thread-like bridges from the PU material have formed across pore edges during the foaming and curing process, which is where most *E. coli* have attached. As the inoculation/growth medium flows through the channels, the planktonic bacteria adhere to these sites, which leads to the assembly of larger colonies. We found no evidence of such activity in SIFs. For PUR-EG foams, fire-retardant polyurethane foams with EG (exfoliating graphite) and APP (ammonium polyphosphate), we noticed that most/all bacteria formed large colonies and almost none on the surface of the cell material (Fig. 11, bottom row). These formations are adjacent to one another. As for PUR, *E. coli* has also adhered to material bridges of the interconnection pores.

## Experimental

### Materials

The foam samples used in the tests were prepared by an injection moulding process. Selected additives were introduced to silicone compositions to assess the effect of additives on bacterial growth in foams, as opposed to a flat non-porous surface.

For SIF synthesis, pre-polymer mixtures were prepared using vinyl- and hydroxyl-terminated poly(dimethylsiloxanes) (5000 cSt), 100% hydrogen-functionalized poly(methyl hydro)siloxane (25–35 cSt), and Karstedt’s catalyst (platinum(0)-1,3-divinyl-1,1,3,3-tetramethyldisiloxane, 0.05% Pt) which were obtained from Hubei Chem, and MilliQ grade water. Moderator SIT7900.0 (1,3,5,7-tetravinyl-1,3,5,7-tetramethylcyclotetrasiloxane) and general strengthening filler HMDZ treated fumed silica SIS6962.0 (LOT# 11698972251) were obtained from Gelest Inc, and muscovite mica from OMYA, Norway (supplied by Virk OÜ, used as received).

**Figure 12:**
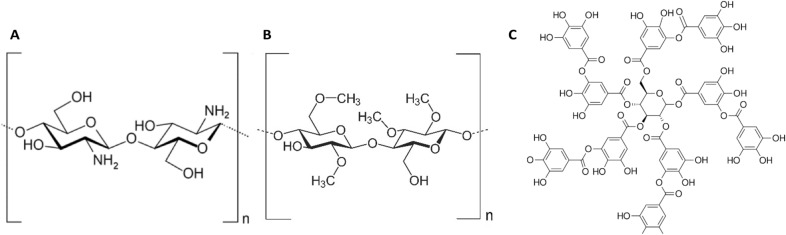
Molecular structures for Chitosan (**A**), methylcellulose (**B**), and tannic acid (**C**).

The resulting open-cell foam densities were in the range of 80–175 kg/m^3^ and were cut into rectangular pieces of 1.3 × 1.4 × 1.5 cm^3^ (volume of 2.7 cm^3^). The porosity of the foam was calculated from the foam density considering the bulk density of the silicone composite was 1 g/cm^3^. As a reference, two polyurethane foams obtained from the industrial production of Estelaxe OÜ were compared using the same method. The list of test foams can be found in Table 1, which is accompanied by structural information on organic additive molecules in Fig. 12.

**Table 1:** Characteristics of the foam samples selected for inoculation with *E. coli* and corresponding additives used for antibacterial assay testing.

Sample group	Foam density (g/cm^3^)	Open volume (%)	Pore diameter, average (mm)	Additive (wt%)	Additive properties			
					Specifications	Surface	Solubility	Considered antibacterial
SIF	0.088	91	0.5 ± 0.1	–	–		–	–
SIF-AC	0.117	88	0.5 ± 0.2	0.9	Activated carbon, < 100 µm, Merck	Hydrophilic	Water-insoluble	No
SIF-SHU	0.150	85	0.2 ± 0.1	0.5	Shungite, carbon content < 50%	Hydrophobic/hydrophilic (carbon in different modifications/metal oxides and silicon oxide)^37^	Water-insoluble /partially soluble	Yes
SIF-CHI	0.135	87	0.3 ± 0.2	0.5	Chitosan, medium molecular weight, 200–800 cps, crystals, Aldrich	Hydrophilic	Water insoluble^34^ (low solubility)	Yes
SIF-MeC	0.110	89	0.5 ± 0.1	5.0	Methylcellulose, < 212 µm (> 95%), Methocel K15MS, Dow	Hydrophilic	Water-soluble	No
SIF-TAN	0.175	83	0.3 ± 0.2	0.5	Tannic acid	Hydrophilic	Water-soluble	Yes
PUR	0.090	91	0.4 ± 0.1^C^	–	–	–	–	No
PUR-EG/APP	0.100	90	0.5 ± 0.2^C^	unknown	^a^Exfoliating graphite (EG)/ammonium polyphosphate (APP), Exolit AP 422 (< 50 µm)	Hydrophobic (EG)/hydrophilic (APP)	Water-insoluble (max. solubility of APP is 0.5 w/w% due to high MW)	Yes^39^/No

^a^Additives in PUR-EG/APP are applied as flame-retardant additives but are not necessarily considered antibacterial in the scope of this work.

^b^Averaged pore diameters were measured and calculated based on SEM images and ImageJ software Fiji application.

^C^Pores in cell walls were excluded from the calculations.

Test organism *Escherichia coli* (*E. coli*) Nissle strain was used in all experiments. Luria–Bertani nutrient broth (LB) and 0.1 M phosphate buffer saline (1XPBS) were prepared by the Institute of Technology, University of Tartu. Formaldehyde solution (37%) for cell fixation was obtained from Panreac AppliChem. Estelaxe OÜ provided the PU-based foam samples, and polysiloxane-based samples were synthesised in-house.

## Methods

### Preparation of polysiloxane foams

The prepolymer mixtures were prepared using a stand-alone mixer with a PTFE-covered rotary blade. The components of pre-mixtures were separated into two distinct parts, one accommodating the catalyst and the moderator, the other containing hydride in addition to other functionalised pre-polymers. Both components were mixed separately, then mixed and injected by a lab-designed injection-moulding device, dispensing a total volume of 500 ml of pre-mixture per foam sample.

### Starting pre-culture

A loop of *E. coli* culture was transferred into the standard LB broth in a glass vial, sealed with a flame-sterilised metal cap. The prepared *E. coli* cell suspension was incubated for 20 h at 37 °C, shaking speed of 180 rpm.

### Inoculation of samples

Before inoculation, a sterilisation of foam samples was carried out in a vacuum oven (Memmert) at 200 °C and 3 mbar for 60 min in the Erlenmeyer flask with an aluminium foil cap as a seal to avoid airborne contamination. A mixture of 30 mL of sterile LB medium and 0.5 mL of *E. coli* pre-culture was prepared for inoculating the foam samples. The initial concentration of the growth medium was approximately (0.5…1) × 10^8^ CFU/ml, determined by plating dilutions and counting colonies in triplicate. The silicone foam used in this research had a low density (85–175 kg/m^3^), small pores (diameter of < 1 mm), and was highly hydrophobic in nature. Thus, it was necessary to de-air the foam samples by immersing and squeezing them in the prepared *E. coli*/LB mixture to allow the bacteria to flow through the open structure. Squeezing was done with sterilised metal tweezers in the vicinity of the flame to kill airborne bacteria. The flasks containing the samples and *E. coli*/LB suspension were shaken for 24 h at 25 °C and 180 rpm.

### Obtaining bacteria from growth medium and foam samples

Firstly, aliquots were collected from the 24-h growth medium surrounding the foam cubes. In addition, a series of washes followed to extract the bacteria from the foam sample. Each foam cube was transferred into a sterile Erlenmeyer flask containing 30 ml of 1 × PBS (phosphate buffer saline solution) and followed by a wash cycle of 10 min at 25 °C and 180 rpm. For further analysis, a sample from each washing (PBS and extracted bacteria) was sampled. This step was repeated until 5 washes were conducted.

### Determining bacterial concentrations

We analysed all initial 24-h growth mediums and subsequent wash mediums to determine the bacterial concentrations. OD_600_ was assessed by a UV/VIS spectrophotometer (Ultrospec 7000, Biochrom) to evaluate the cell density of the wash samples. The samples were serially diluted tenfold in each step using 1 × PBS. 100 µL of dilutions were plated onto the LB Agar plate and incubated overnight at 37 °C. The bacterial colonies were counted, and initial concentrations were calculated considering specific dilution factors. The antibacterial activity/efficacy can be seen by the decreased ratio of bacteria considering the bacterial concentration of the foamless inoculum.

### Visualising the bacterial adhesion in cells via SEM

For each specific composition, a set of foam cubes were removed from inoculation suspensions after 24 h. The cubes were immersed in 3.7% formaldehyde in 1 × PBS solution for cell fixation and kept at +4 °C for further analysis. A stepwise exchange of the FA/PBS solution in the foam's pores for ethanol (99.5%) was done, e.g. serial dehydration. The foam samples were kept in each solution for a minimum of 2 h — in 40, 50, 60, 70, 80, 90, and 96 vol% of ethanol. In addition, overnight in 99.5 vol% ethanol, and another 99.5vol% for storage.

The supercritical CO_2_ extraction process, a necessary step for preparing cells for imaging, was conducted using a critical point dryer (E3100, Quorum Technologies) and thermostat (Proline RP 1845, LAUDA). For imaging the cross-sections under SEM (Hitachi TM3000, 15 kV), the samples were freeze-cut using a scalpel and sputter-coated with a 7.5 nm layer of gold.

### Elemental mapping of polymer composite surfaces

SEM–EDX technique was applied to map the elemental distribution and acquire the surface composition of foam materials. For EDX, SwiftED3000 (Oxford Instruments) was utilised in combination with SEM (Hitachi TM3000). Elemental compositions were analysed by collecting data from 20 points, and the results were averaged.

### Contact angle determination

The hydrophilicity of the foam materials was evaluated by measuring the contact angle formed between water droplets and the surface of the polymer foam and the skin-like layer which forms upon moulding. For this purpose, drops of water were mounted on three different areas. Results are the mean value of three measurements on different polymer film parts. Results are summarised in the Supplementary S1.

## Conclusions

Antibacterial activity for different silicone foams compared with polyurethane foams was evaluated by inoculating the foams with gram-negative *E. coli*, one of the most common pathogenic organisms found in mattresses and seat cushions. Different fluids, usually accompanied by microorganisms, are prone to enter the material's pores during the repetitive compression that characterises their application. Therefore, the quantitative method we have applied is suitable for describing the bacterial growth in elastic and three-dimensional structures in conditions where an excess carrying medium is available and the planktonic bacteria are free to adhere to the surface.

We focused on comparing the antimicrobial activity of the commercially available and low-cost natural additives integrated into the polymer matrix, as it is somewhat cumbersome to immerse or dip-coat the end product. The following conclusions from the results of this study can be drawn:

- While the majority of *E. coli*'s outer membrane is hydrophilic, the combination of partial hydrophobicity and SIF surface microroughness is sufficient to allow the attachment of the bacteria;
- The antimicrobial effect, or the lack of it, could be explained by the thin polymer layer covering the additive particles expected to act as antibacterial sites;
- Although the additive particles are covered with a thin silicone layer, the water-insoluble hydrophilic additives incorporated into pre-polymers affect the *E. coli* attachment on the surface of the foam by increased surface roughness;
- Water-soluble additives, such as tannic acid, show a considerable antibacterial effect when dissolving from the polymer matrix;
- During the 24-h incubation period, the gram-negative bacteria *E. coli* is more prone to adhere to polysiloxane-based elastomer surface than to polyurethane-based foam. However, the bacterial concentration in the surrounding medium of the pristine polysiloxane is lower than for a standard polyurethane.

We conclude that using low-cost natural additives without dip-coating but initial incorporation into the polymer matrix makes it possible to avoid microbial biofilm formation on the surface of silicone foams. For future research, it would be essential to analyse the variations in filler content in the range where the mechanical properties of the elastomer are acceptable for a desired application.

## Supplementary Information

Supplementary Information.

## Data Availability

The datasets supporting the conclusions of this article are included within the article and its supplementary files. The additional SEM images used and/or analysed during the current study are available from the corresponding author upon reasonable request.
