# Supplementary material for: Microbial growth and adhesion of Escherichia coli in elastomeric silicone foams with commonly used additives
Source: Sci Rep. 2023 May 26;13:8541. doi: 10.1038/s41598-023-35239-9 (PMC10220030; doi:10.1038/s41598-023-35239-9)
Supplement: Supplementary file 1 — Supplementary Information. [file 41598_2023_35239_MOESM1_ESM.pdf]

Microbial growth and adhesion of *Escherichia coli* in elastomeric silicone foams with commonly used additives

Ingrid Rebane *[a], Hans Priks [a], Karl Jakob Levin [a], İsmail Sarigül [a], Uno Mäeorg [b], Urmas Johanson [a], Peeter Piirimägi [c], Tanel Tenson [a], and Tarmo Tamm [a]

[[a] Institute of Technology, University of Tartu, Nooruse 1, 50411 Tartu, Estonia, [b] Institute of Chemistry, University of Tartu, Ravila 14a, 50411 Tartu, Estonia, [c] Estelaxe OÜ, Estonia, correspondence to: Ingrid Rebane, ingrid.rebane@ut.ee]

Keywords: silicone foam, polyurethane foam, *Escherichia coli*, antimicrobial activity, microbial growth, PDMS, polysiloxane, biofilm formation, wound dressings

SUPPORTING MATERIAL

Filler particles and wall cross-sections

From the cross-sections of the pore walls in **Figure S 1**, we can observe filler particles (e.g. general filler flakes) and antibacterial additive agglomerated particles. Although there are differences in the thickness of the walls and the amount of used fillers (SIF vs PUR), we can distinguish the particles distributed all over the matrix, not concentrated on the surface of the pore. A standard strengthening filler and agglomerated additive particles were revealed after the freeze-cut process, visible in pore cross-sections and the pore walls underneath a thin polymer layer.

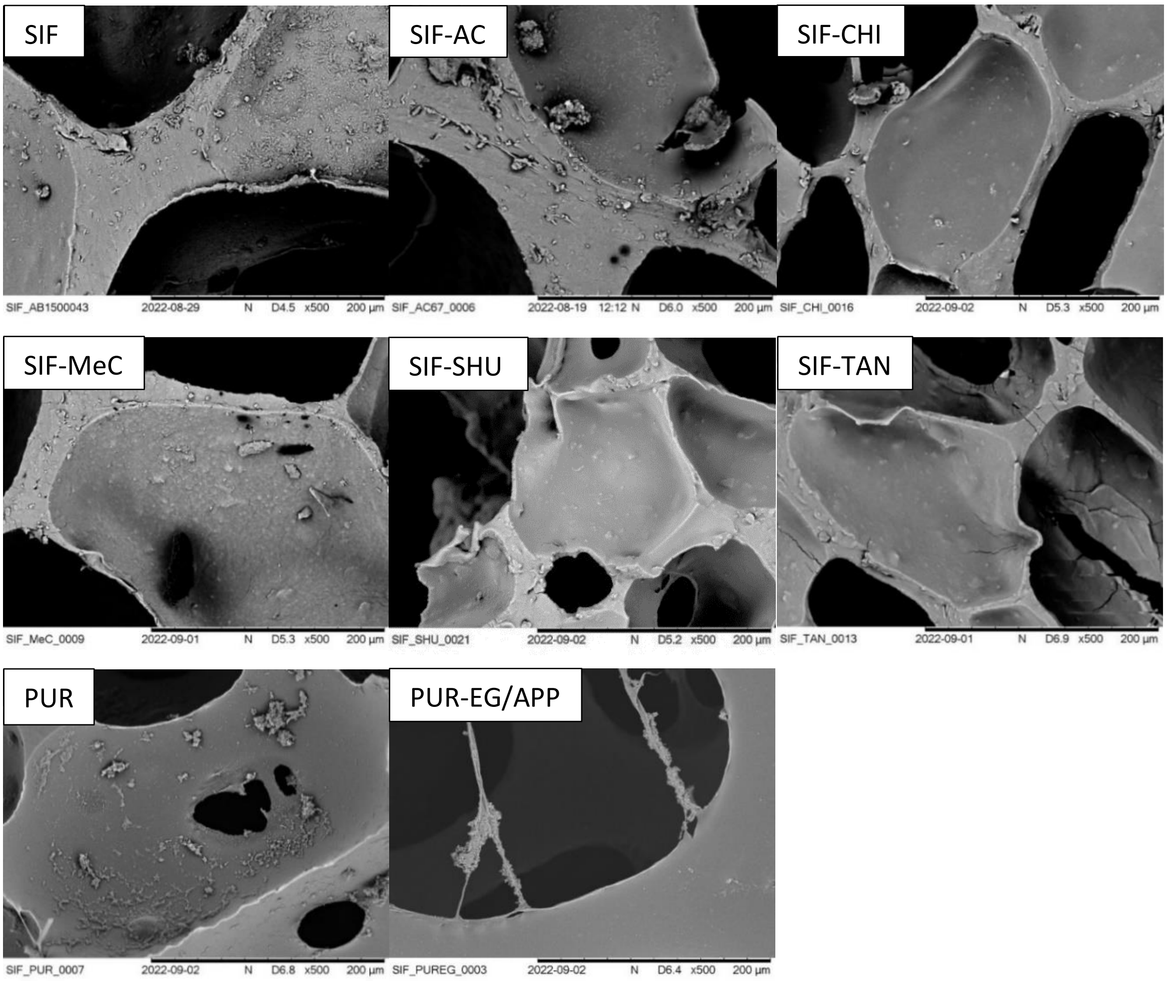

Figure S 1. *SEM images*: *Cross-sections of foam pores and pore walls.*

Please do not adjust margins

Foam structures for SIF and PUR

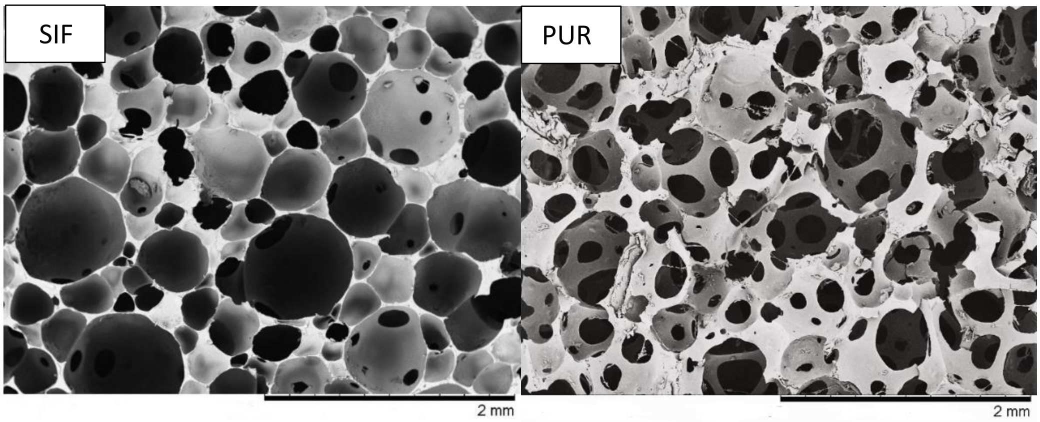

Figure S 2. *Comparison of structures. The surface area for SIF (left) is comparably larger than for PUR (right).*

Contact angle values

Table S 1. *Contact angle values of selected foams show good hydrophobicity (>90°) of the surfaces – both for open pore foams and skin-like monolithic surfaces.*

Specimen		Mean (deg)	Mean deviation (deg)
**SIF**	Skin-like	107.5	1.0
	open foam	112.5	0.3
**SIF-AC**	Skin-like	101.8	0.2
	open foam	112.7	1.1
**PUR**	Skin-like	106.5	0.3
	open foam	103.9	0.3
**PUR-EG**	Skin-like	105.1	0.2
	open foam	99.9	0.3

Table S 2. *Concentrations of E. coli colony forming units (CFU) in growth medium (LB, ml) and washing medium (PBS)* resulting from two serial dilutions.

E. coli, CFU/ml	After 24 h	SE, after 24 h	Wash I	SE, Wash I	Wash II	SE, Wash II	Wash III	SE, Wash III	Wash IV	SE, Wash IV	Wash V	SE, Wash V
SIF	1.01E+09	5.39E+08	5.79E+07	2.35E+07	1.52E+07	7.50E+06	2.60E+06	1.05E+06	2.64E+06	5.31E+05	3.43E+06	1.22E+06
SIF-AC	3.31E+09	1.07E+09	1.71E+08	4.31E+07	5.91E+07	1.44E+07	9.61E+06	3.78E+06	4.70E+06	4.86E+05	1.93E+07	7.51E+06
SIF-MeC	1.12E+09	5.08E+08	3.65E+07	1.37E+07	1.33E+07	3.89E+06	6.91E+06	2.20E+06	9.64E+06	1.87E+06	2.79E+06	6.26E+05
SIF-SHU	6.29E+08	3.29E+08	5.02E+06	2.50E+06	5.74E+05	3.09E+05	6.32E+05	4.37E+05	5.65E+05	2.72E+05	4.60E+05	2.37E+05
SIF-CHI	2.73E+08	1.78E+08	1.02E+06	2.66E+05	1.06E+06	6.60E+05	1.51E+05	1.06E+05	7.70E+04	4.54E+04	9.82E+04	4.35E+04
SIF-TAN	1.58E+08	1.06E+08	2.58E+06	8.94E+05	5.32E+05	2.98E+05	1.85E+05	7.13E+04	1.25E+05	2.64E+04	1.18E+05	4.79E+04
PUR	2.97E+09	7.91E+08	3.41E+08	4.25E+07	1.89E+07	5.20E+06	4.93E+06	1.84E+06	7.68E+05	1.36E+05	2.60E+05	1.84E+05
PUR-EG/APP	1.64E+09	3.18E+08	6.16E+08	5.58E+07	4.88E+07	9.95E+06	5.04E+06	1.44E+06	2.97E+06	5.04E+05	9.77E+05	1.02E+05
SIF 0h	9.48E+06	1.81E+06	3.29E+05	7.98E+04	2.52E+04	6.67E+03	2.00E+03	4.38E+02	2.17E+03	7.85E+02	3.01E+03	1.14E+03
SIF 24h	1.60E+09	3.47E+08	1.63E+08	4.66E+07	4.75E+07	1.73E+07	3.11E+07	1.38E+07	8.35E+06	4.10E+06	8.50E+06	6.02E+06
FLASK	1.73E+09	7.46E+08	2.89E+08	7.85E+07	1.03E+07	2.25E+06	6.47E+06	9.87E+05	3.09E+06	1.65E+06	2.66E+06	1.07E+06
AIR	no medium	no medium	9.66E+07	1.60E+07	1.36E+07	6.84E+06	8.69E+06	4.67E+06	6.68E+06	4.52E+06	9.34E+06	7.57E+06
